# Farming strategies of 1^st^ millennium CE agro-pastoralists on the southern foothills of the Tianshan Mountains: A geoarchaeological and macrobotanical investigation of the Mohuchahangoukou (MGK) site, Xinjiang, China

**DOI:** 10.1371/journal.pone.0217171

**Published:** 2019-06-05

**Authors:** Yuqi Li, Michael Storozum, Duo Tian, Michael Frachetti, Kai Su, Xin Wang

**Affiliations:** 1 School of History, Nanjing University, Nanjing, Jiangsu, China; 2 Institute of Archaeological Science, Fudan University, Shanghai, China; 3 School of Cultural Heritage, Northwest University, Xi’an, Shaanxi, China; 4 Institute of Middle Eastern Studies, Northwest University, Xi’an, Shaanxi, China; 5 SAIE Laboratory, Department of Anthropology, Washington University in St. Louis, St. Louis, MO, United States of America; 6 Department of Anthropology, Washington University in St. Louis, St. Louis, MO, United States of America; 7 Hejing County Office for the Preservation of Ancient Monuments, Hejing, Xinjiang, China; Ohio State University South Centers, UNITED STATES

## Abstract

Archaeological evidence emerging over the past decade clearly illustrates that agro-pastoralists living along the foothills of major mountain chains in Central Asia (the so-called “Inner Asian Mountain Corridor” or IAMC) facilitated the spread of domesticated grains through their direct involvement in farming. While the environmental conditions across the northwestern slopes of the IAMC provided adequate resources for incipient farming and herding as early as the mid-3^rd^ mill. BCE, the development of local agricultural strategies on the extremely arid and eroded foothills on the southeastern, leeward side of the mountains remain comparatively less studied. Our study tackles this problem by combining geoarchaeological analysis with conventional macrobotanical identification in the investigation of a 1^st^-mill. CE agro-pastoralist farming site, Mohuchahangoukou (MGK), located on the arid foothills of the Tianshan range. Our results illustrate how ancient agro-pastoralists at MGK innovated irrigation systems both to combat water shortage and, importantly, to trap sediments carried by flood-water for crop cultivation. By synthesizing currently available data, we estimate that they managed to trap about 40 cm of fine-grained sediment within a span of 200 years or even less. These stone-built field systems helped water a diverse stand of crops and create deeper soils in an otherwise deflated landscape with thin desert soils. Since we detected high levels of salt concentration (>2 dSm^-1^) in the lower portions of all three test trenches we analyzed, we conclude that soil salinization might have affected the long-term sustainability of this form of irrigated field management. We also infer that, besides engineering efforts, the ancient agro-pastoralists at MGK had to resolve the scheduling conflicts between irrigated farming and animal herding through labor specialization.

## Introduction

**Fig 1:**
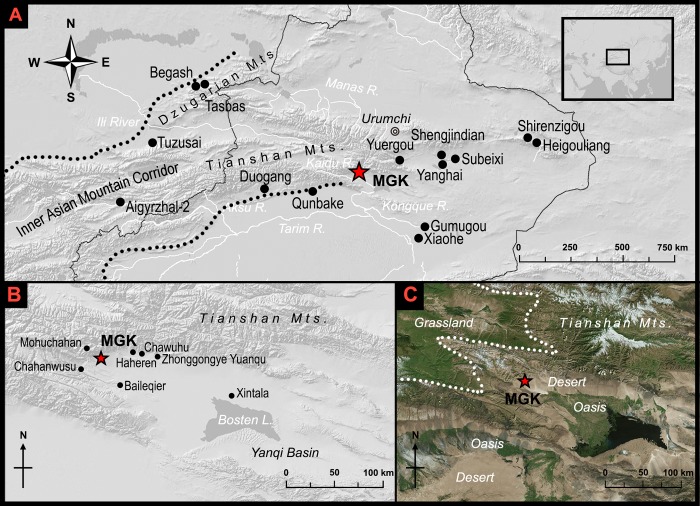
Location of MGK. (A) Location of MGK and other sites mentioned or cited in this article (except for those in the Yanqi Basin). (B) Important archaeological sites in the Yanqi Basin. (C) Ecozones in the Yanqi Basin area.

Recent research in the heartland of Central Asia shows that east-west connectivity was deeply rooted in the interaction networks that formed among mountain agro-pastoralists by the mid-3^rd^ mill. BCE [1,2]. Since the early Bronze Age, mobile herding communities occupied the foothill territories of the so-called “Inner Asian Mountain Corridor” (henceforth IAMC), a mountainous zone extending from the Hindu Kush to the Tianshan (and Altai) Mountains [1,3–7]. In light of the ecological opportunities of vertical pasture distribution documented across the IAMC, archaeological evidence and ethnographic studies suggest that mobile pastoralists have long used a range of highland pastures in the summer and dispersed to lower-elevation camps in the winter [8–10]. This broad trend in pastoralist mobility has subsequently been linked to material and information transfers, eventually leading to the formation of extensive interaction networks along the IAMC and beyond [2]. Within such interaction networks, the flow of materials and information shaped diverse transmissions of pastoralism as well as the spread of domesticated grains and farming practices [11–18] (Fig 1A).

We now also know that Bronze Age pastoralists, or more precisely agro-pastoralists, engaged in farming along the IAMC, on both the relatively humid and fertile windward side of the mountains and, more surprisingly, the hyper-arid leeward southeastern side since the start of the 2^nd^ mill. BCE [3,4,19–24]. Given the harsh environment and the perceived logistical incompatibility between mobile herding regimes and irrigated farming, the agricultural landscapes created by ancient agro-pastoralists to cultivate the arid foothills of NW China offer an essential case study for understanding the transformation and variability of ancient domesticated economies across Inner Asia.

Increased research into the farming strategies of Eurasian agro-pastoralists accompanied the recent discovery of domesticated grains along the IAMC [11,17,25], but have remained focused on the well-watered northwestern slopes of the Central Asian mountain systems. However, more in-depth research is needed to understand the emerging strategies for agriculture on the leeward side of Inner Asia’s mountain rain shield, most importantly because of the steep increase in aridity as one moves eastward into Xinjiang. Although the overall environment is still demanding for crop cultivation on the windward side, the mountain foothills contain microenvironments more suitable for dry-land crops. Using hardy crops and cultivation strategies such as multi-cropping, agro-pastoralists could often guarantee a portion of harvest would survive under these unpredictable rainfall regimes [21,25,26]. On the leeward side, however, niche exploitation was far from enough to ensure farming success as the local agro-pastoralists faced even harsher climatic conditions and a general lack of topsoil on the mountain foothills. More sophisticated cultivation and possible niche construction strategies are assumed [27], but their specific forms remain to be explored.

**Fig 2:**
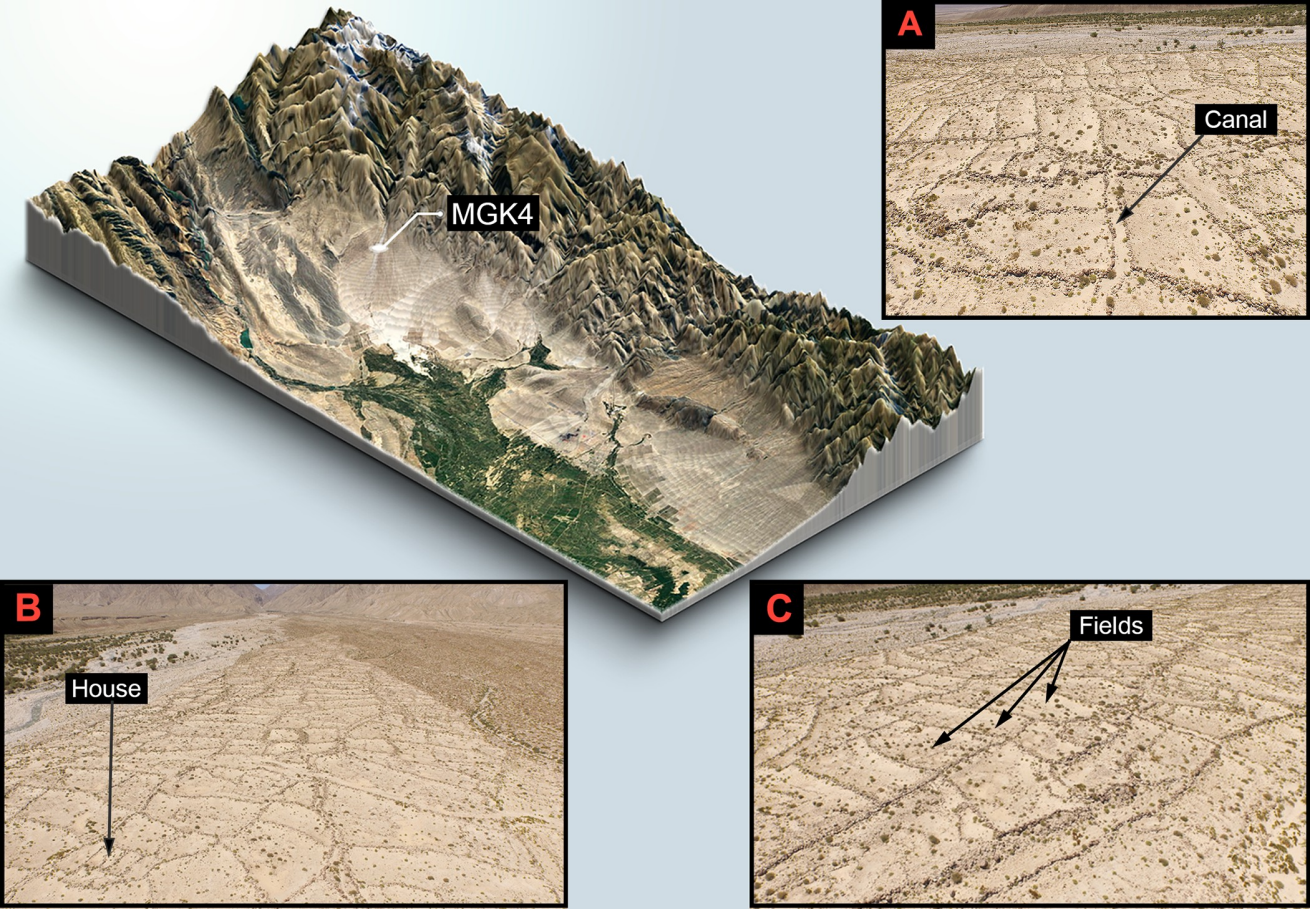
Geographical setting of MGK4 and aerial photos of the site. (A) Aerial photo of MGK4 (from east to west). (B) Aerial photo of MGK4 (from southeast to northwest). (C) Aerial photo of MGK4 (from southeast to northwest).

In this study, we address this issue by combining conventional test excavations and macrobotanical identification along with geoarchaeological analyses to study irrigation and other farming strategies at Mohuchahangoukou (henceforth MGK) [28,29]. MGK is large agro-pastoralist farming landscape in the Yanqi Basin of Xinjiang, China, consisting of seven concentrated site clusters measuring more than 200 ha. in total (Fig 2). The focus of this paper, MGK4, is directly dated by AMS to the early 1^st^ mill. CE.

Although researchers have reported a range of agro-pastoralist sites in southern Xinjiang, MGK provides an excellent location to investigate the farming strategies of ancient transhumant agro-pastoralists. Better-known early agricultural sites in this region, such as Gumugou [30], Xiaohe [31], and Xintala [32], probably were associated with more sedentary lifeways and are located relatively far from the mountains. Whether their inhabitants practiced seasonal vertical transhumance, the basis of the agro-pastoralist interaction networks that facilitated the spread of early agriculture along the IAMC, remains a question [33,34]. Agro-pastoralist sites located in foothill environments offer ample botanical evidence of agriculture such as Chawuhu [35], Yanghai [24], and Duogang [36], yet the remains of fields and farming strategies are not available, making it impossible for us to reconstruct many aspects of ancient farming strategies beyond the grain types themselves. MGK is a unique agro-pastoralist site because of its location on a mountain foothill that contains a well-preserved ancient agricultural landscape [37]. Investigating MGK allows us to explore ancient farming strategies from the perspective of soils.

Here, we present the excavation results of five test trenches, geoarchaeological analyses of three soil columns, and the macrobotanical identification of five flotation samples. These data provide a multi-variate analysis of desert soils from different contexts and a rare archaeobotanical dataset for the early 1^st^ mill. CE in southern Xinjiang (for other examples see [38,39]). Based on the results of these analyses, we argue that irrigation systems were key technologies used by seasonally mobile agro-pastoralists to farm the arid environment of the southern Tianshan foothills. Their channels and field systems were designed to effectively trap sediment and channel water, taking advantage of seasonal flood and runoff dynamics. The trapped sediments provided a necessary soil substrate for crops to grow, and their field constructions allowed greater water retention in arid periods of the year. The downside of this mode of irrigated farming is that they might lead to severe soil salinization problems in the long run. Other than the engineering efforts, ancient agro-pastoralists probably balanced the conflicting labor and mobility requirements of irrigated farming and animal herding through labor specialization. This study represents the first comprehensive investigation of an agro-pastoralist farming site on the leeward side of the IAMC.

### Regional setting

The Yanqi Basin is located on the south of the Tianshan Mountains in southern Xinjiang (Fig 1B). From the Tianshan to the bottom of the Yanqi Basin, there are three major ecozones, the mountains, the foothills, and the oasis (Fig 1C). The mountains have a cool and relatively moist climate due to their high elevations. Glacial melt and summer rains feed dozens of rivers that originate in the mountains and drain into the basin. The highland meadows in the mountains and intermontane valleys serve as the main summer pasturelands for modern-day pastoralists (pers. obser.). Occupying two major highland basins, the Bayinbuluke Grassland is the largest pastureland in this zone.

The foothills comprise a series of alluvial fans and their adjacent stony desert. Compared to the mountains, the climate on the foothills is also cool but drier. Abundant cobble- and boulder-sized alluvial and colluvial deposits cover the ground surface. Erosion scars on granite boulders and a lack of topsoil indicate strong wind deflation. In the open area, sparse xerophytic vegetation dots the landscape. Other less drought-tolerant plants cluster in thin bands along the braided river channels. Except for some pastoralist winter camps, no settlements are found on the foothills. On the other hand, the lowland oasis with its deep, fertile soils and easy access to river water, has a long history of a dense population despite its hyper-arid climate (annual rainfall around 76.3 mm according to [40])[41,42]. Overall, the mountains and the oasis are suitable for herding animals and irrigated agriculture, respectively. Local pastoralists set up winter camps on the foothills, which has limited agricultural potential without significant technology to overcome aridity and soil erosion.

### Paleoclimate

Bosten Lake in the southwest of the Yanqi Basin preserves one of the best Holocene climate records in Xinjiang. Many paleoclimatologists have reconstructed the regional Holocene climate using lake sediments from Bosten Lake. Although not without exceptions, their work generally shows that the early 1^st^ mill. CE was warm and dry. Zhong and Shu found that the water level of Bosten Lake lowered while its salinity increased during this period [43]. Wünnemann et al. identified that Bosten Lake had a negative water balance from 50 to 900 CE, indicating a dry climate [44]. Even though Huang disagrees that the entire early 1^st^ mill. CE was a dry period, he agrees that the late phase of this period, when MGK was most likely occupied, was very arid [45]. Overall, the paleoclimate data allow us to argue that ancient agro-pastoralists adopted these farming strategies under harsh conditions.

### Site description and archaeological context

MGK is located in an alluvial fan at the northern rim of the Yanqi Basin and belongs to the administrative region of Hejing County, Xinjiang Uygur Autonomous Region of China. The entire site consists of seven zones (MGK1 through MGK7), all of which are distributed along the Mohuchahan River, a perennial stream originating from the Tianshan [37]. Its discharge remains low throughout the year, but regularly experiences flash floods during the summer months that bring a large amount of sediment down from its upper reaches.

**Fig 3:**
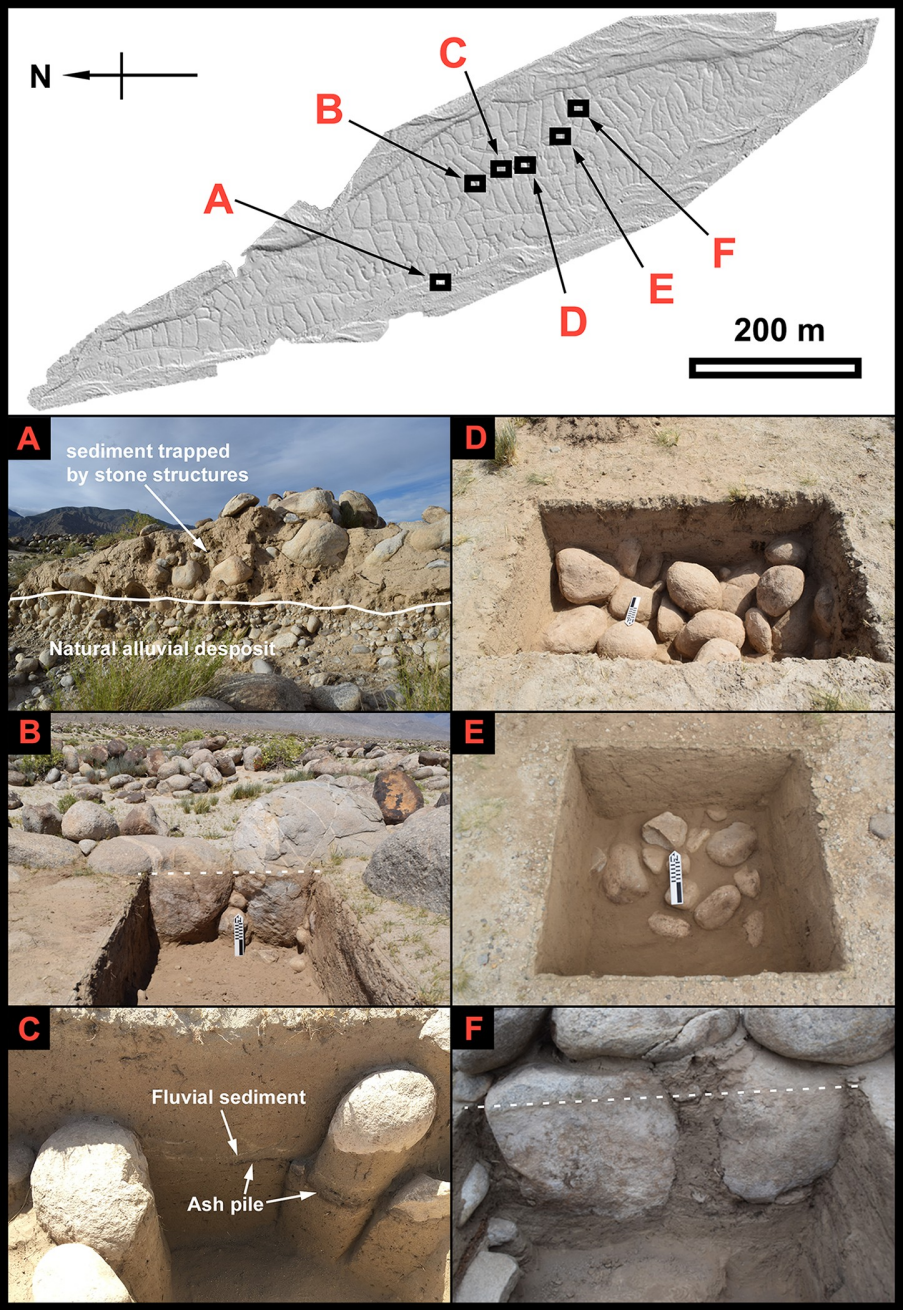
Digital elevation model of MGK4 and the investigated locations of this study. (A) Western profile of the MGK4 terrace. (B) the eastern boulder wall of T1-House 2 after excavation. (C) the northern profile of T3-Canal 1 after excavation. (D) T4-Cistern 1 after excavation. (E) T5-Field 1 after excavation. (F) the western profile of T2-House 3.

Due to the imposing size of MGK (circa 200 ha), excavation fieldwork has focused on MGK4. This zone qualifies as a good starting point for its medium size (circa 25 ha), clear boundaries, and dense surface stone features (Figs 2 and 3). Its western and eastern sides are bounded by the Mohuchahan River and one of its fossil channels respectively. Sitting on a northwest-southeast running terrace, MGK4 is 0.5 to 2 m higher than nearby river banks. From its northwestern to the southeasternmost point, MGK4 measures 1.6 km long and 260 m wide in the middle and gradually tapers off toward the two ends. The elevation of MGK4 ranges from 1810 m asl at its northwestern end to 1730 m asl at its southeastern end.

Previous surveys and test excavations suggest that MGK4 was built by an agro-pastoralist community dating from the early 1^st^ mill. CE [37]. Archaeologists have identified houses, burials, and a rather complex irrigation system consisting of check dams, canals, cisterns, and field boundaries at MGK4 (Fig 3). Besides the architectural remains, surface finds such as an iron plow and two chipped stone hoes demonstrate the site’s connection with ancient farming. The main line of material evidence for pastoralism at the site is a large number of sheep/goat bones and dung found within house contexts. MGK4’s proximity to the Bayinbuluke Grassland in the Tianshan Mountains and modern pastoralists’ routine use of the site area to set up winter camps also provide ethnographic parallels for mobile pastoralist strategies in the area. Based on three radiocarbon dating results, Li et al. tentatively placed MGK4 to the third and fourth century CE [37].

The history of foothill farming by agro-pastoralists in the Yanqi Basin area has been traced back to the late Bronze Age (2500–900 BCE), but MGK is the only confirmed ancient farming site with this agricultural tradition (another site that still awaits confirmation is Haheren, see [46], Fig 1B). Archaeologists have retrieved botanical remains of crops from both Chawuhu (cal. 1000–500 BCE) and Baileqier (cal. 392 BCE-CE 21), but no fields have been found associated with these two agro-pastoralist cemeteries [35,47,48]. From other late Bronze Age to early historic period foothill sites, such as Mohuchahan (Phase 1, 1210–780 cal. BCE), Zhonggongye Yuanqu (circa 400 BCE-CE 1) and Chahanwusu (circa 700 BCE-CE 400), archaeologists have recovered grinding stones, mortars, and sickles possible used for harvesting crops [49–51], but still have no evidence of fields.

The textual record suggests that the Wusun, an agro-pastoralist group, controlled the Tianshan area north of the Yanqi Basin during most of the Han dynasty (202 BCE—CE 220) [41,52]. Since the Wusun are known for farming on many mountain foothills of western Central Asia [21,25], we infer they probably had fields on the Tianshan foothills. The discovery of MGK qualifies as the best representation of a two-thousand-year-old agro-pastoralists farming tradition.

## Materials and methods

### Geoarchaeological and archaeobotanical sampling

**Fig 4:**
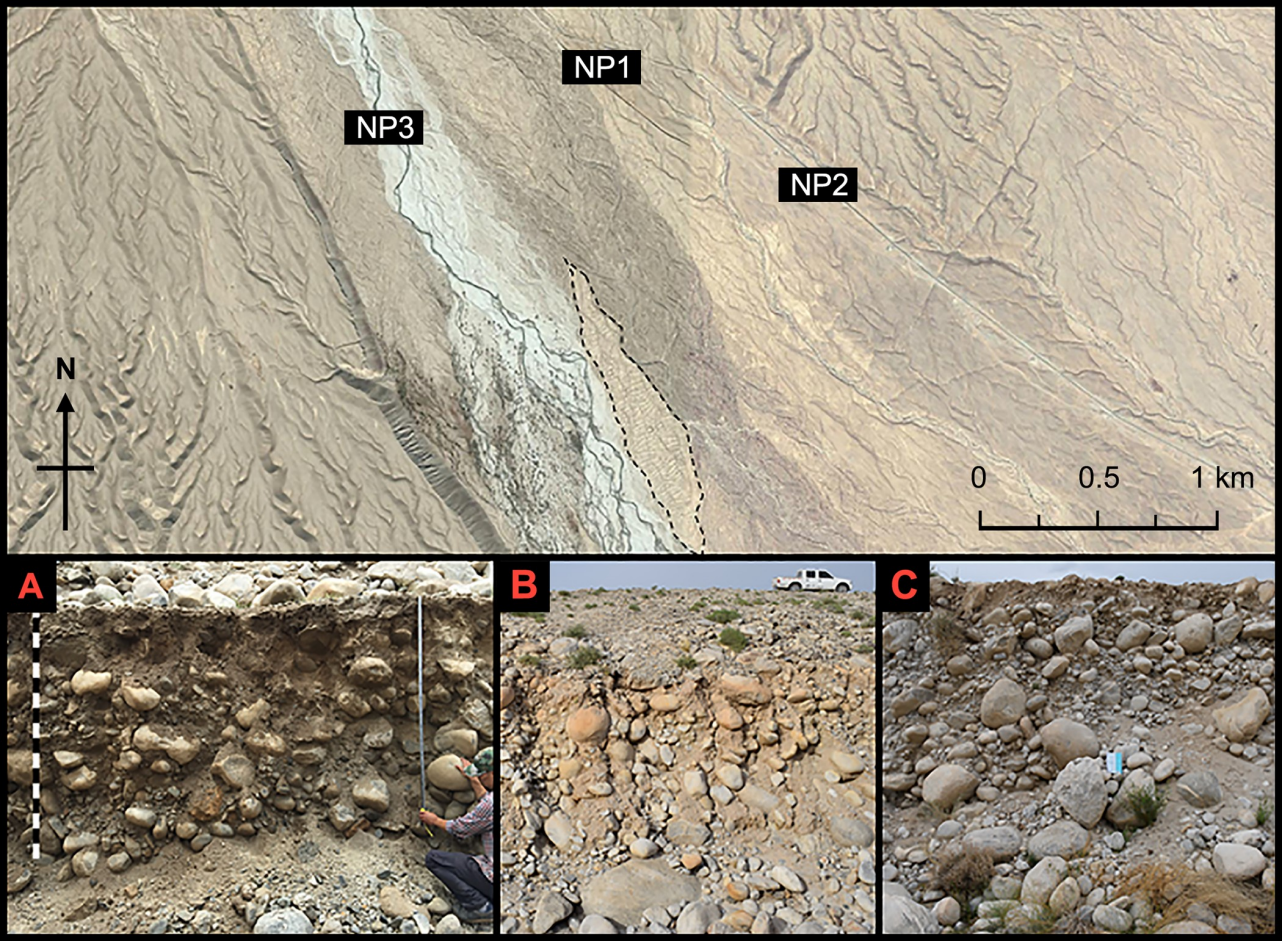
The three natural profiles examined in this study. (A) Natural profile 1. (B) Natural profile 2. (C) Natural profile 3.

We first examined the western profile of the terrace (Fig 3A) to understand the stratigraphy of MGK4 (42°24'46.41"N, 85°46'48.37"E). Then, we compared this profile with three off-site natural profiles (NP1, NP2, and NP3) near MGK4 to evaluate the impact of farming and occupation on the local soils (Fig 4). Afterward, we excavated five test trenches to investigate four types of features, houses (T1-House 2 and T2-House 3), canals (T3-Canal 1), cisterns (T4-Cistern 1), and fields (T5-Field 1) to further examine the stratigraphy and collect scientific analysis samples (Fig 3). Additionally, we conducted a salvage excavation at a looted burial, which we assigned as Burial M2.

In total, we collected 34 bulk soil samples for geoarchaeological analysis, of which nine from T1-House 2 (T1-House 2–1 through T1-House 2–9, 0–45 cm; all depths were measured from top to bottom), 15 from T2-House 3 (T2-House 3–1 through T2-House 3–15, 0–75 cm), and 10 from T5-Field 1 (T5-Field 1–1 through T5-Field 1–10, 0–50 cm). The sampling procedure involved excavating the test trenches to sterile natural alluvial deposits and taking a sample (circa 200 g) every five centimeters in a column. We intentionally included samples from the natural alluvium in the bottom to use them as reference samples. Throughout the test excavations, we strove to collect representative AMS radiocarbon dating samples for every important context (e.g., the lowest context and the contexts that contain hearths).

Flotation samples were taken from the ash piles and hearths we encountered during the test excavations. Of the five flotation samples we took (SS1 through SS5), four (SS1 through SS4) came from T2-House 3, and the other one, SS5, came from T3-canal.

This project was approved by the Hejing County Office for the Preservation of Ancient Monuments, which was directly involved in this study. We confirm that our field studies did not involve endangered or protected species.

### Geoarchaeological analyses

We conducted six different types of analyses, including particle size analysis, sequential loss on ignition, magnetic susceptibility, pH, electrical conductivity (EC), and elemental geochemistry. Each of these analyses generates data on proxies useful for detecting the impact of ancient farming practices on the local soils.

Particle size analysis, also known as grain size analysis, measures the size of different particles that constitute sediment samples. Researchers commonly use it to determine the environment and energy associated with deposition processes [53,54]. In this study, we use particle size analysis to investigate whether irrigation and other farming practices had a significant influence on local sedimentation process.

Sequential loss on ignition measures the content of organic matter (OM) and calcium carbonate in bulk soil samples. OM is an essential component of soils, critical to soil functions and fertility. Many forms of human land use activities, such as tillage, burning, manuring, and mulching can lead to changes in soil OM content [55–57]. Measuring the OM content in bulk soil samples has the potential to identify ancient land use practices. Most calcium carbonate forms on the surface of soils and then is gradually transported to different depths in arid to semi-arid regions [58]. It usually has a positive relationship with soil pH values. Researchers often use it for soil age estimation and paleoenvironment reconstruction [59–61]. In this study, we used calcium carbonate as a proxy to detect human interference in the local soil formation process.

Magnetic susceptibility is a bulk signal that measures the combined magnetic behaviors of different minerals in soils [62]. Regional variations in soil magnetic susceptibility are mainly determined by geology and soil processes, but local variations may be attributed to factors such as land use, burning, and pollution [63]. Following this principle, archaeologists use low-frequency (LF) magnetic susceptibility (or simply magnetic susceptibility) and frequency-dependent (FD) magnetic susceptibility to detect ancient human activities [62,64]. The former, measured at 0.46 kHz, reflects the magnetic susceptibility of all minerals. The latter is the difference between low- and high-frequency (4.6 kHz) magnetic susceptibilities [62]. It is particularly sensitive to superparamagnetic (SP) minerals, which are very fine (smaller than 0.03 μm) particles, often related to fire use.

The pH analysis measures the acidity or alkalinity of soils and presents its result in the form of pH values. Since acidity or alkalinity of soils has a direct impact on plant nutrition availability, pH values are often used as an indicator of soil health [65]. While the pH values of natural soils are mainly determined by the mineral compositions of their parent materials, the pH values of anthropogenic soils may be significantly influenced by human activities, such as irrigation, burning fuel, and depositing trash. We mainly use pH values to infer the impact of ancient irrigation practices on the soil health at MGK4.

The soil electrical conductivity (EC) test measures the salinity of soils [66]. Since improper irrigation often leads to an increase in soil salinity in arid regions, we use EC values to evaluate the impact of ancient irrigation practices on the soil salinity at MGK4.

Geochemical analysis detects ancient human activities by comparing the chemical compositions of anthropogenic and natural soils [67–69]. In a recent article, Oonk et al. summarized 17 elements commonly used as proxies for human activities in archaeological soils, including P, B, Ni, Se, Ba, Na, Ca, Fe, Cu, Pb, Zn, K, Mn, Sr, Rb, Th, and Cs [68]. We have measured the concentration of all these elements in the soil samples except for P, B, Rb, and Cs. We have also applied principal component analysis (PCA) to identify the elements that have the least correlation with other elements. The rationale is that human activities rather than parent materials of soils are likely to be responsible for their “abnormal” distribution patterns.

We conducted the particle size analysis, sequential loss on ignition, magnetic susceptibility analysis, pH analysis, and EC test at the Geoarchaeological laboratory of Washington University in St. Louis, and the geochemical analysis at the Nano Research Facility of the same university. All the bulk samples were air dried before any laboratory procedure was applied. Particle size measurement was conducted with a Micromeritics Saturn II DigiSizer laser diffraction system. No pretreatment was applied except for removing large organic inclusions by hand as described in Gale and Hoare [70]. The classification of the sediment particles followed the protocols developed by the United States Department of Agriculture. Particles less than 0.002 mm were defined as clay, between 0.002 and 0.05 mm as silt, and between 0.05 and 2 mm as sand. Samples for sequential loss on ignition were first weighed and then burned at 550°C for four hours, following the protocol by Nelson and Sommers [71]. The samples were weighed again and then burned at 1000°C for 2 hours. We calculated the percentage of calcium carbonate following the protocols by Heiri, Lotter, and Lemcke [72]. We measured the high and low magnetic susceptibility of the samples with a Bartington MS2B magnetic susceptibility sensor and calculated the frequency dependence [62]. Soil samples used for pH analysis and EC test (50 g each) were mixed with water at a ratio of 1:2. We measured the soil extract with a pH meter and an EC meter and calculated the EC at 25°C following the protocols by Bado et al. [73]. The geochemical analysis was conducted using a Perkin Elmer Elan DRC II ICP-MS. After these analyses, we stored the rest of the soil samples at the Geoarchaeology Laboratory of Washington University in St. Louis.

**Fig 5:**
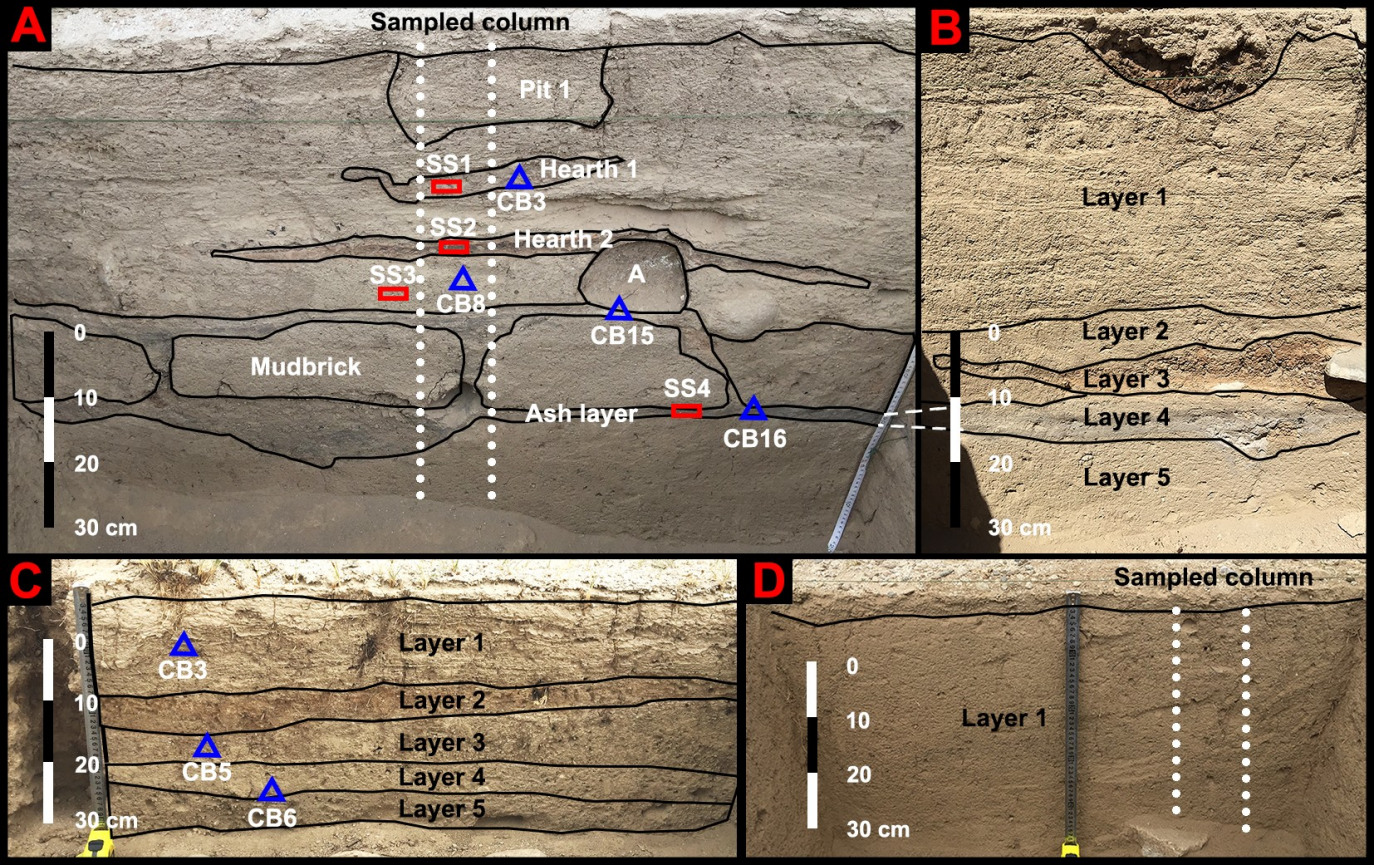
Test trench profiles examined in this study. (A) the northern profile of T2-House 3. (B) the eastern profile of T2-House 3. (C) the southern profile of T1-House 2. (D) the western profile of T5-Field 1.

Additionally, we sent nine AMS samples from a total of 24 for AMS dating (Table 1, Fig 5). Three of them, respectively from T2-House 3, T3-Canal, and Burial M2, were processed by the DirectAMS laboratory. All the other six were processed by the Woods Hole Oceanographic Institution. Three of these six samples came from T1-House 2, while the other three came from T2-House 3.

**Table 1:** Radiocarbon dates from MGK4.

Sample code	Lab code	Material	Depth (cm)	Context description	Radiocarbon age / BP	Calibrated date (2σ range)^a^
M2-CB1	D-AMS 012434	Charcoal	115	burial M2	2618±32	834–771 cal. BC
T1-CB3	OS-131607	Charcoal	7.5	House 2, Layer 1	1720±20	253–304 cal. AD; 312–387 cal. AD
T1-CB5	OS-131609	Charcoal	27.2	House 2, Layer 3	1670±20	335–418 cal. AD
T1-CB6	OS-131608	Charcoal	41.3	House 2, Layer 4	1,800±15	136–254 cal. AD; 301–316 cal. AD
T2-CB3	OS-131610	Charcoal	18.0	House 3, Layer 1, Hearth 1	1,720±20	253–304 cal. AD; 312–387 cal. AD
T2-CB8	OS-131611	Charcoal	37.3	House 3, Layer 1, Hearth 3	1,590±20	416–537 cal. AD
T2-CB15	OS-131612	Charcoal	58.2	House 3, Layer 1, beneath the stone boundary of hearth 3	1,680±20	264–274 cal. AD; 331–413 cal. AD
T2-CB16	D-AMS 018451	Charcoal	63.2	House 3, Layer 4	1709±27	252–305 cal. AD; 311–397 cal. AD
T3-CB1	D-AMS 018452	Charcoal	78.0	Canal 1, Sediment fillings	1665±30	258–283 cal. AD; 323–429 cal. AD; 496–506 cal. AD

^a^The authors calibrated all dates with Oxcal v4.2.4; r:5; intCal 13 atmospheric curve.

### Macrobotanical analysis

The flotation samples were floated in the Hejing County Office for the Preservation of Ancient Monuments, using the simple bucket method described in Fritz [74], Pearsall [75], and Watson [76]. The soil was broken down using water separation by means of manual agitation. Samples were measured by pouring soil in 0.5 L increments into a self-made measuring device. The recorded volumes were rounded up to the nearest integers or half-integers. After agitation, the suspended organic materials were decanted in a 0.2 mm geological sieve until no more buoyant material was observable. The light fraction material was then transferred to a muslin pouch for drying in the shade. The non-buoyant residue was not processed for a heavy fraction. The laboratory analysis was conducted in the Archaeobotany Laboratory at Northwest University in Xi’an, China, following the protocols described by Zhao [77]. Due to the existence of a large number of modern plant roots in the light fraction, the initial step was to remove all visually non-carbonized material. The rest of the light fraction was then passed through a set of nested mm geological sieve. All botanical material larger than 2.00 mm was sorted as one unit and weighed, while the rest of the samples were broken down into units using sieves of 1.0 mm, 0.7 mm, and 0.5 mm. Material smaller than 0.335 mm was left unprocessed and labeled “pan.” Carbonized seeds presented in S1 Table were sorted from the sieve units and systematically analyzed afterward. These seeds are currently stored at the Archaeobotanical Laboratory of Nanjing Normal University.

## Results

### Western profile of the terrace and nearby natural profiles

From the western profile of the MGK4 terrace, we identified two distinct layers (Fig 3A). The lower layer is an alluvial deposit full of mixed-size gravels and cobbles. The upper layer mostly consists of fine-grained sediments, but it also has some inclusions of large cobbles and boulders. The complete absence of gravel-sized sediments leads us to believe that the upper layer is not a poorly-sorted natural deposit, but was rather formed through human actions.

All the natural profiles we examined (NP1 through NP3) have several layers of gravel and cobble-rich alluvial deposit (Fig 4). None of them contains thick layers of fine-grained sediments like the upper layer we saw in the western profile of the MGK4 terrace.

### T1-House 2

We identified five layers, Layer 1 through Layer 5, beneath 5 cm of topsoil at T1-House 2 (Fig 5A). Material evidence of ancient human occupation is found throughout the test trench except for Layer 5. Surface finds include several pieces of highly degraded sandy red- and gray-ware and oxidized iron fragments. The topsoil contains sheep/goat bones and teeth. Layer 1 is a yellowish layer constituted of laminated sediments, suggesting low energy alluvial deposition. We retrieved a single piece of charcoal that dated to 253–378 cal. CE (T1-CB3) and a small number of sheep/goat bones from this layer. Layer 2 is sandy and loose in texture and reddish in color. We only found a single piece of sandy red-ware from Layer 2. Layer 3 is a charcoal-rich layer with a slightly dark gray color. The retrieved materials from Layer 3 include oxidized iron fragments, sheep/goat ribs, scapulae, and teeth. An AMS sample places Layer 3 to 335–418 cal. CE (T1-CB5). Layer 4 has a light yellowish color, which is in contrast with the color of Layer 3. We retrieved a piece of sandy red-ware, a hemispherical stone spindle whorl, some sheep/goat bones, and several pieces of charcoal from Layer 4. An AMS sample provides a chronological range of 136–316 cal. CE for this Layer 4. Layer 5 only has subtle differences from Layer 4 in color, but its texture is much coarser. We found lots of cobbles and coarse sand in Layer 5, but no charcoal or artifact, suggesting that Layer 5 is a sterile alluvial deposit.

The test excavation also exposed two abutting boulders that constituted part of an internal wall of House 2 (Fig 3B). One of them sits at 40 cm below the modern ground surface, while the other sits at 46 cm below. The narrow gap between them was carefully filled with fist-sized cobbles. Judging from the shape of the gap, we believe that the cobbles were inserted from a side of the wall after the two boulders were lined in position first. Thus, it is unlikely that the ancient constructors of House 2 dug trenches to place these boulders. Instead, they must have placed the boulders on the ground surface then. Following this line of reasoning, we infer that the thick sediments at this location were mostly accumulated after the construction of House 2.

**Fig 6:**
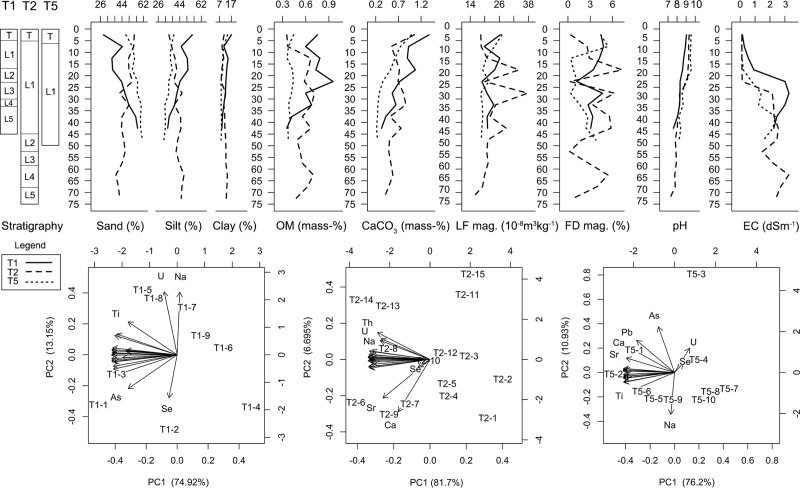
Results of geoarchaeological analysis. (Note: 1. In the graph of the stratigraphic sections, letter T refers to topsoil, while letter L refers to Layer. 2. In the second row of graphs, the overlapping element names were omitted in the scatter plot of the PCA results).

The particle size analysis suggests that the sediments in T1-House 2 largely has a coarse to fine distribution pattern from the bottom to top (Fig 6). Since the laminated beds indicates that these sediments are mostly alluvial deposits and suggests the water flow gradually slowed over time. Aside from natural changes to the local hydrology, the constructed field boundaries and other stone features are the most likely factor to account for the decreased flow of water.

The concentration of OM is generally low, averaging 0.65 mass-% (σ = 0.17, n = 9), but it corresponds well with our interpretation of the stratigraphy (Fig 6). The peak value occurs at the depth of 20–25 cm, which matches with the charcoal-rich Layer 3. The lowest value comes from the naturally formed Layer 5. The topsoil has a slightly higher OM content, reflecting the influence of vegetation growth and grazing activities. The content of calcium carbonate gradually increases from the bottom to the top of the soil column. Correspondingly, the pH values increase almost steadily from 7.59 to 9.03. This is consistent with a previous study that reports the pH values of the topsoil in northern Hejing County are generally over 8, with a high of nearly 10 (Ren et al. 2010).

The magnetic susceptibility analysis shows the ancient occupation did not leave strong signatures in the soils (Fig 6). The LF magnetic susceptibilities are generally low, averaging 22.46*10^-8^m^3^kg^-1^ (σ = 2.57, n = 9). The three peak values occur at the depth of 0–5 cm, 10–20 cm, and 30–35 cm. The FD magnetic susceptibilities vary within a narrow range from 1.18 to 4.78 with an average of 3.53 (σ = 1.09, n = 9). The single extreme outlier is the lowest value of 1.18 at the depth of 20–25 cm. The overall low magnetic susceptibilities and their fluctuations across the soil column suggest the ancient occupation was ephemeral and probably punctuated, which is consistent with the mobile lifestyle of agro-pastoralists.

The EC test indicates that ancient irrigation might have incurred soil salinity problems at MGK4. The EC values of soils from the lower portion (20–45 cm) of the soil column are consistently over 2 dSm^-1^, signaling an excessively high level of salt concentration [78](Fig 6). We attribute the origin of these salts to ancient irrigation because MGK4 is relatively immune to naturally occurring soil salinization (primary salinity). The MGK4 area has deep low-salinity groundwater and a sloped topography [67]. These characteristics make primary salinity unlikely to occur at MGK4. The cause of the salinity at MGK4, hence, could only be attributed to human activities (secondary salinity). Given the extensive irrigation facilities at the site, we infer that improper irrigation caused the salting. As for the medium level of salt concentration in the soils from the upper portion (0–20 cm) of the soil column, we believe it is likely a result of the leaching effect of rainfall after the site was abandoned [78].

The geochemical analysis shows that the 13 elements we analyzed mostly have strong correlations with each other, except for U, Se, and Na (Fig 6). Of the three elements, U is a trace element not commonly associated with ancient human activities. Se can be an indicator of ancient human habitation, but it does not seem to be enriched in this case the analysis shows its content remains stable throughout the soil column. Only Na appears to be enriched, most likely as a result of improper irrigation.

### T2-House 3

From T2-House 3 we have identified five layers beneath the topsoil, Layer 1 through Layer 5 (Fig 5C and 5D). Except for Layer 5, each of these layers contains material evidence of ancient human activities. Surface finds include several pieces of sandy red- and gray-ware. The loose and sandy topsoil contains oxidized iron fragments and sheep/goat dung. Layer 1 is comprised of yellowish laminated alluvial deposits. It encapsulates a pit (Pit 1) and three charcoal-rich hearths (Hearth 1 through Hearth 3). Two of the three hearths, Hearth 1 and 2, are identifiable from the profile. The other one, Hearth 3, does not leave any trace on the profile because it centers at the middle of the test trench. Nevertheless, these three hearths still roughly stack upon each other. Only thin layers of sediments separate them. While Hearth 1 and 2 only have shallow pits, Hearth 3 has associated stone boundaries. Besides charcoal, we have also retrieved sheep/goat bones from Hearth 2 and 3. An AMS sample (T2-CB3) dates Hearth 1 to 253–387 cal. AD. Another AMS sample (T2-CB8) from Hearth 3 dates to a much later period, 416–537 cal. AD. Since we found some rodent holes underneath Hearth 3 during the excavation, we suspect that bioturbation might have contributed to the reverse chronology.

Layer 2 distinguishes itself from Layer 1 by a lack of laminated sediments. It contains several horizontally lined mudbricks and their surrounding fills. The fills contain ash, charcoal, and sheep/goat bones. A charcoal sample (T2-CB15) sandwiched by the stone boundary of Hearth 3 and the mudbricks gives us a date of 264–413 cal. AD.

Layer 3 is a thick patch of animal dung with a brown to orange color, while Layer 4 is an ashy stratum underlying Layer 3. The well-preserved pellets allowed us to attribute them to sheep/goats [79]. Since the dung is found in a house context and has an associated hearth and ash, we infer that it was used as fuel by ancient inhabitants of MGK4. Similar practices have been reported in many ancient and modern Central Asian pastoralist societies that lack access to wood resources [80–82]. An AMS sample dates Layer 4 to 252–397 cal. AD.

Layer 5 has a relatively homogenous sandy sediment deposit and a yellowish color. Since it does not contain any artifacts or ecofacts, we concluded that Layer 5 represents a natural alluvial deposit.

The test excavation also reveals that the boulder walls of House 3 extend deep into the ground (Fig 3F). One of the boulders sits at a depth of 48.5 cm, while the other at a depth of 43.0 cm. The positions of these two boulders suggest that the majority of the alluvial sediments at T2-House 3 was accumulated after the construction of the boulder walls.

The sediment particle sizes largely show a fine to coarse trend from the bottom to the top of the soil column (Fig 6), suggesting the water flow that deposited the sediments gradually speeded up over time. If the natural conditions kept stable, it is probably mainly due to the impact of the irrigation system.

The sequential loss on ignition and pH tests show that ancient habitation had limited impact on the local soils. OM content of the soils is generally low, averaging 0.57 mass-% (σ = 0.11, n = 15) (Fig 6). Except for the two samples from the uppermost 10 cm, the rest of the samples all share similar OM content. From the bottom to the top of the soil column, the pH values steadily increase from 7.59 to 9.03. Neither of the two proxies indicates the influence of ancient human activities. Only the slight fluctuation of the calcium carbonate content across the soil column likely reflects some human influence.

The magnetic susceptibility analysis shows the ancient habitation at T2-House 3 spanned several episodes (Fig 6). The LF magnetic susceptibilities average 22.83 10^−8^ m^3^ kg^-1^ (σ = 5.96, n = 15). Their three peak values occur at the depth of 15–20 cm, 25–30 cm, and 40–45 cm. Each of these peaks likely corresponds to an occupation episode, while the troughs between them represent gaps between occupation episodes. The FD magnetic susceptibilities, averaging 3.52 (σ = 1.09, n = 15), show an additional peak at the depth of 60–65 cm, which corresponds to the ash in Layer 4.

The EC test shows a salt concentration pattern very similar to what we have seen at T1-House 2 (Fig 6). The soils in the lower trench (25–75 cm) have excessively high salt concentration, while those from the upper trench (0–25 cm) have very low levels of salt content. Following the same line of reasoning, we infer improper irrigation also led to the accumulation of an excessive amount of salt in the soils at T2-House 3.

The geochemical analysis shows that the content of all the elements we measured appear to have close correlations with each other (Fig 6). Only Ca and Sr seem to have relatively different distribution patterns from other elements. Both of these two elements are established proxies for ancient human activities. Our analysis shows that they were both enriched largely in the same depth, where traces of ancient human activities were identifiable from the stratigraphy.

**Fig 7:**
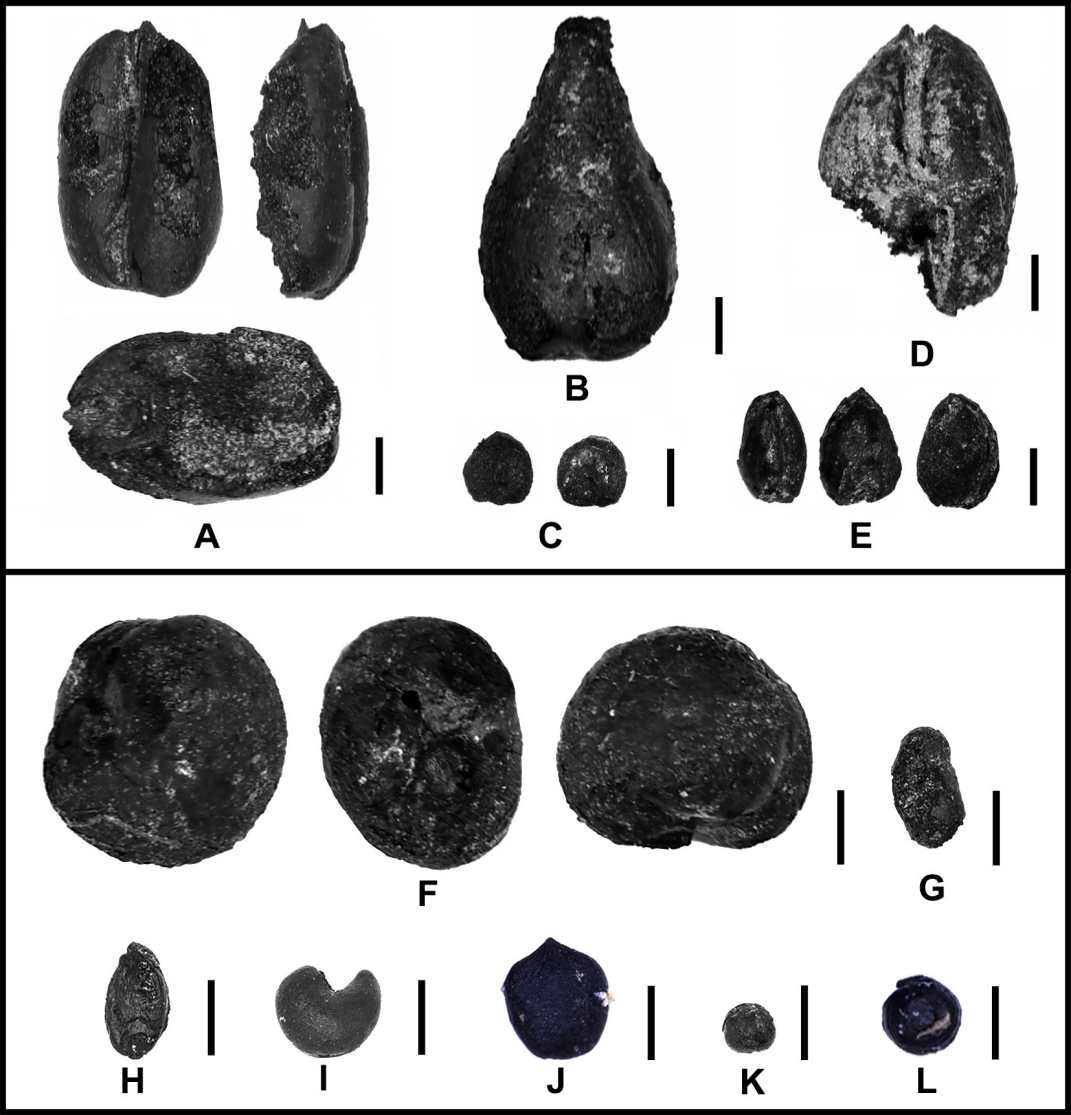
Examples of charred seeds retrieved from MGK4. A) *Triticum aestivum*; B) *Vitis vinifera*; C) *Setaria italica*; D) *Hordeum vulgare*; E) *Panicum miliaceum*. F) Fabaceae; G) *Trigonella*; H) *Setaria viridis*; I) *Malva* sp.; J) *Persicaria* sp.; K) *Vaccaria* sp.; L) *Chenopodium*.

From the four flotation samples collected at T2-House 3, we have retrieved a grape pip and four types of domesticated grains (Fig 7), free-threshing wheat (*Triticum aestivum*), naked barley (*Hordeum vulgare*), broomcorn millet (*Panicum miliaceum*), and foxtail millet (*Setaria italica*). Additionally, we identified a wide variety of wild plants (Fig 7), such as *Chenopodium*, *Malva* sp., and *Setaria* sp (S1 Table).

### T3-Canal 1

Before our excavation, loose sand and some ephedra (*Ephedra sinica*) covered the ground surface. After removing the loose sand, we found a thick layer of sandy and homogenous sediments that extends to about 80 cm deep (Fig 3C). The only visible feature from the profile is a thin lens of water-lain sediment at about 40 cm deep, suggesting that the canals used to be much deeper. Right beneath this sediment lens is a charcoal rich ash pile, probably resulting from cleaning the canals by burning shrubs and other woody plants. The sediments adjacent to the ash pile also contains many pieces of charcoal. The most deeply buried charcoal sample we encountered and dated was T3-CB1 (258–509 cal. AD) from 78 cm below the modern ground surface.

Through archaeobotanical analysis, we retrieved wheat and wheat/barley grains and many wild seeds from the ash pile (Fig 7). Detailed information about the macrobotanical identification can be found in S1 Table.

### T4-Cistern 1

The stratigraphy of T4-Cistern 1 lacks artifacts or ecofacts, but it shows strong characteristics of alluvial deposition. We found 40 cm of laminated sandy deposit below the loose sand and scanty vegetation-covered ground surface. We also found a possible artificially laid cobble layer in the bottom of the test trench (Fig 3D). The cobbles largely have similar sizes and appear to align in rows. Larger excavation units are needed to confirm their anthropogenic nature.

### T5-Field 1

T5-Field 1 has a very simple stratigraphy. The artifacts and ecofacts we found in this test trench are almost all concentrated on the surface and the bottom of the trench. From the surface, we have collected sandy red- and gray- wares and iron fragments. Underlying these artifacts was a thin sterile topsoil layer. Below the topsoil, we encountered over 40 cm of yellowish homogeneous sediment deposit, which we named Layer 1 (Figs 3E and 5B). Unlike the stratigraphic sections we observed at other locations of MGK4, Layer 1 lacks any trace of the laminated structure. The reason probably was due to the disturbance of tillage [83]. In the very bottom of Layer 1 and right above the sandy and cobble-rich natural alluvial deposit beneath this layer, we found some tiny bone fragments and two pieces of highly degraded ceramic sherds. We infer almost all the soils in our test trench were formed after the habitation started at MGK4.

The sediment particle size gradually turns finer from the bottom to the top of the soil column (Fig 6), suggesting the water flow that deposited the sediment gradually slowed down. Since this particle distribution pattern contrasts with that of T2-House 3, we infer the construction of the irrigation system probably led to many localized variations in surface hydrology at MGK4.

The sequential loss on ignition shows that the concentration of carbonate calcium gradually increases from the bottom to the top of the soil column (Fig 6). It also shows that all the samples have almost identical OM content, averaging only 0.40 mass-% (σ = 0.03, n = 10). Neither of these two proxies indicates strong influence from ancient human activities.

The magnetic susceptibility analysis suggests that human activities started to affect the soils at T5-Field 1 before the formation of the fields, but they remained at a low intensity throughout the occupation phase. The early appearance of human activities at the site is reflected in the small peak of FD magnetic susceptibility near the bottom of the test trench. Between the depths of 30 to 50 cm, the FD magnetic susceptibility increases steadily with the depth, indicating the existence of burned remains at the bottom of the trench. The low intensity of ancient human activities is reflected in the generally low magnetic susceptibility of the soils, which only averages 20.53*10^−8^ m^3^ kg^-1^ (σ = 2.72, n = 10).

The EC test shows the soils from the lower portion of the soil column generally have high to excessively high levels of salt concentration, while those from the upper portion have a much lower level of salt concentration (Fig 6). This pattern echoes what we have seen at T1-House 2 and T2-House 3. Following the same line of reasoning, we think improper irrigation led to severe soil salinity problems at MGK4.

The geochemical analysis shows that Na, U, Se, and As are four elements that have very different distribution patterns from other elements we analyzed. Of these four elements, U and As usually are not regarded as elements associated with ancient human habitation or farming activities. It is likely that their distribution patterns were mainly determined by certain natural conditions. As in other test trenches, the concentration of Se also remains stable throughout the soil column. It is unlikely that Se has been enriched by human activities in this case. Therefore, only Na appears to be influenced by ancient human activities, most likely through improper irrigation.

## Discussion

Based on our analyses at MGK4, we argue that ancient agro-pastoralists on the leeward side of the IAMC used irrigation systems to trap sediments to improve their local farming conditions. Our test excavations at MGK4 reveals that before people occupied the site, they first cleared the area of the cobbles and boulders that covered the landscape. Then they constructed the irrigation system and used sediment trapping as an effective method to create and regenerate fields. Irrigation systems designed with similar intentions, in fact, are common in many other parts of the world. For example, ancient Near Eastern sites, such as Tepe Gaz Tavila (5400–4800 BC, Iran) [84], Wadi Faynan (Bronze Age to Roman period, Jordan) [85], Jawa (Early Bronze Age, Jordan) [86], and Petra (Nabataean/ Roman, Jordan) [87], all have similar stone-constructed irrigation systems. However, sediment trapping practices have never been reported at agro-pastoralist sites in the mountains of Inner Asia.

Although the irrigation system recovered at MGK4 likely improved local farming conditions in the short term, our results also show that it might have caused soil salinization problems over time. In natural conditions, the area around MGK4 should be relatively immune to soil salinization problems because it is located in the upper alluvial fan where the water table was relatively low, and the groundwater has low salt concentration. Its sloped topography should have made it even less prone to such problems. However, our EC tests detected high levels of salt concentration in the lower portions of T1-House 2, T2-House 3, and T5-Field 1. The geochemical analysis also shows that Na has been enriched in the soils at both T1-House 2 and T5-Field 1. The construction of the irrigation systems created numerous basins on the ground surface. They were effective at retaining water, but they could have also caused drainage problems and eventually led to soil salinization. Ancient agro-pastoralists growing crops with this form of irrigation systems probably had to shift fields after cultivating at a location for certain periods of time to achieve long-term sustainability.

Our investigation of MGK4 also challenges previous understandings of the relationship between ancient agro-pastoralists on the leeward side of the IAMC and the environment. Earlier studies on this topic generally depict ancient agro-pastoralists as niche dwellers who tend to practice low-investment agriculture in ecologically rich areas [24]. Our study, however, shows that ancient agro-pastoralists were also active niche constructors. They invested substantial amounts of efforts to build the irrigation system and to improve the farming conditions at MGK4. The extensive stone structure visible on the site surface only represent a fraction of the labor requirement of the project. The test excavations at T1-House 2 and T2-House 3 suggest the stone structures may all have a good portion being buried underground. We argue that ancient agro-pastoralists on the arid foothills of the Tianshan significantly modified their landscape to promote farming within a multi-strategy system of pastoralism [88].

In addition to this irrigation technology, we also suggest that ancient agro-pastoralists adopted social strategies such as labor specialization to ensure farming success in this precarious environment [25]. Labor specialization within or between households was the most likely way inhabitants at MGK could reconcile the logistical and seasonal differences inherent to their mixed economy with mobile animal herding and farming. In the local context, agro-pastoralists had to herd their animals on highland pastures in summer–simply because summer pastures in lowland contexts are effectively non-existent. However, the summer crops they cultivated at MGK, such as grapevines, wheat (most likely spring wheat as farmers in the Yanqi Basin only started to grow winter wheat in recent years [89]), foxtail, and broomcorn millet, all needed to be watered at the same time under the hyper-arid climate of the Yanqi Basin. Their irrigation system also needed to be attended as summer was the critical time for the system to channel water and to trap sediments. For these reasons we conclude that some members of the group must have remained on site year-round to maintain the irrigation system. In such systems, some sectors of the community may emerge as farming specialists, while others might be more focused on animal herding (e.g Yomut Turkmen, [90]). From a global perspective, both textual record and ethnographic research suggest that labor specialization based on gender, age, kinship, and other factors are rather common among pastoralist groups [91–93].

## Conclusion

Within the broad field of Eurasian archaeology, researchers are focusing more attention on the agro-pastoralist interaction networks along the IAMC, which arguably laid the geographic and social foundation for what is later called the “Silk Road”. Botanical evidence suggests that these networks formed on the basis of pastoralist seasonal mobility on both sides of the IAMC and facilitated the spread of early agriculture. However, current research has yet to provide a satisfying explanation of the specific farming strategies adopted by ancient agro-pastoralists to counteract the harsh environment on the leeward side of the IAMC. We have combined test excavations and macrobotanical identification with geoarchaeological analyses to show how ancient agro-pastoralists used irrigation systems for effective crop cultivation within a diversified economic strategy. Through the construction of an irrigation system, ancient agro-pastoralists appeared to have trapped around 40 cm of fine-grained sediment within 200 years or less. In a landscape that lacks topsoil, they created the soils for the fields they farmed and effectively watered them. This form of irrigated agriculture helped them achieve short-term farming successes but may have resulted in possible salinization problems. This argument is supported by the high levels of salt concentration (>2 dSm^-1^) we detected in the lower portions of T1-House 2, T2-House 3, and T5-Field 1. As a result of soil salinization, the ancient agro-pastoralists probably had to shift fields on a regular basis to achieve long-term sustainability. Our results also show that due to the strong labor and mobility conflicts between irrigated farming and vertical animal herding in summer, ancient agro-pastoralists had to rely on labor specialization to engage in both subsistence strategies at the same time.

## Supporting information

S1 Table Macrobotanical remains identified from the flotation samples of MGK4. (XLSX)

S2 Table Results of geoarchaeological analysis (except for geochemical analysis). (XLSX)

S3 Table Results of geochemical analysis. (XLSX)

S1 Fig Oxcal plot of radiocarbon dates. (TIF)
