# Supplementary figures and images for: Farming strategies of 1st millennium CE agro-pastoralists on the southern foothills of the Tianshan Mountains: A geoarchaeological and macrobotanical investigation of the Mohuchahangoukou (MGK) site, Xinjiang, China
Source: PLoS One. 2019 Jun 5;14(6):e0217171. doi: 10.1371/journal.pone.0217171 (PMC6551202; doi:10.1371/journal.pone.0217171)

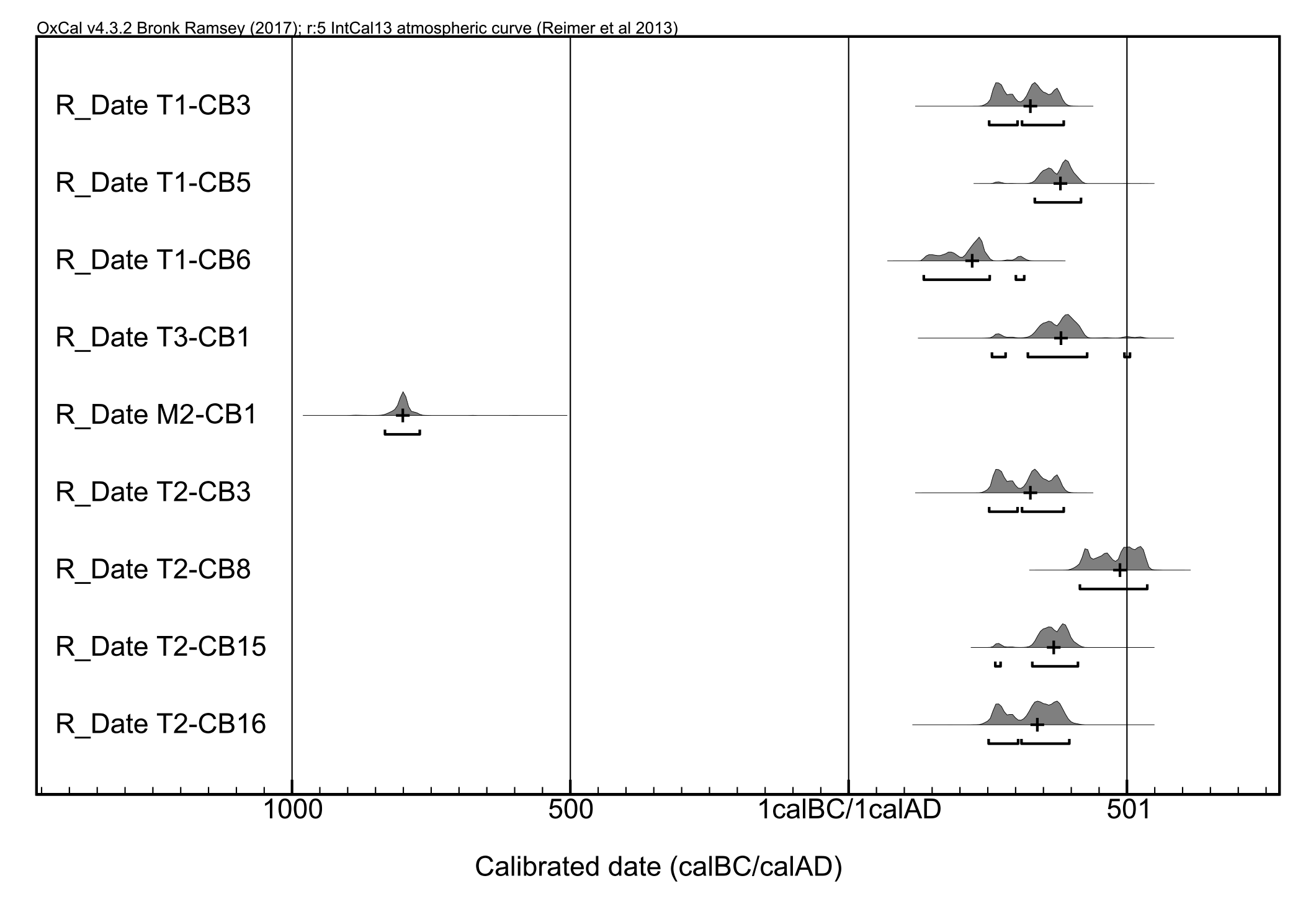

Supplement: S1 Fig — (TIF) [file pone.0217171.s004.tif]
